# Prevalence of Subclinical Peripheral Neuropathy in Patients With Autoimmune Connective Tissue Disorders: An Observational Cross-Sectional Study

**DOI:** 10.7759/cureus.70294

**Published:** 2024-09-26

**Authors:** Mariraj Indiran, Priyadharshini Venugopalan, Gowrishankar Arumugam

**Affiliations:** 1 Internal Medicine, Saveetha Medical College and Hospitals, Saveetha Institute of Medical and Technical Sciences, Saveetha University, Chennai, IND

**Keywords:** autoimmune connective tissue disorders, carpal tunnel syndrome, peripheral neuropathy, sjögren's syndrome, steroid use

## Abstract

Background

Autoimmune connective tissue disorders (AICTDs) are a group of chronic inflammatory diseases characterized by immune responses targeting the body's own tissues. Peripheral neuropathy, which can be symmetrical or asymmetrical, is a common complication in AICTD patients. Early detection of subclinical peripheral neuropathy remains challenging, as nerve conduction studies are not routinely performed. This study aimed to determine the prevalence of subclinical peripheral neuropathy among patients with AICTD and to assess its association with potential predictor variables, including laboratory parameters and autoimmune markers.

Materials and methods

This observational cross-sectional study included 100 patients undergoing treatment for AICTDs for over three years, conducted over 15 months. Ethical approval and informed consent were obtained before detecting subclinical peripheral neuropathy through nerve conduction studies on bilateral ulnar, radial, sural, peroneal, and tibial nerves at a tertiary-level medical college hospital. Inclusion criteria encompassed patients treated at the tertiary hospital, not on immunosuppressants other than steroids, with at least three years of treatment, while exclusions were made for established peripheral neuropathy and other specified conditions. Descriptive statistics, Chi-square/Fisher’s exact tests, independent samples t-tests, and logistic regression were used for data analysis at a 0.05 significance level, with IBM SPSS Statistics for Windows, Version 20 (Released 2011; IBM Corp., Armonk, NY, USA).

Results

Subclinical peripheral neuropathy was present in 18% (n = 18) of the study participants, while 82% (n = 82) did not exhibit neuropathy. Patients with subclinical neuropathy had a significantly higher mean age (49.66 ± 4.77 years) compared to those without neuropathy (46.02 ± 12.7 years, p = 0.047). The majority of the study population was female, 79 (79%), and there was no significant gender difference between the groups (p = 0.513). There was no significant association between inflammatory markers (C-reactive protein (CRP), erythrocyte sedimentation rate (ESR)) and neuropathy. However, patients with subclinical neuropathy were more likely to test positive for rheumatoid factor (RF) (8 (66.7%) vs. 71 (58.5%), p = 0.011) and anti-cyclic citrullinated peptide (Anti-CCP) antibodies (11 (78.5%) vs. 32 (47%), p = 0.032). Steroid use did not significantly affect the presence of neuropathy (p = 0.769).

Conclusion

The study found a high prevalence of subclinical peripheral neuropathy in AICTD patients, detectable only by nerve conduction studies. RF and Anti-CCP antibodies were associated with subclinical neuropathy, suggesting these markers could guide early detection, and inform treatment intensification to reduce neuropathy prevalence among AICTD patients.

## Introduction

Autoimmune connective tissue disorders (AICTDs) are a collection of long-lasting inflammatory autoimmune diseases triggered by antibodies or T-cell responses that target the body's antigens [1]. Connective tissue diseases (CTDs) encompass a range of conditions, including systemic lupus erythematosus (SLE), Sjögren syndrome (SS), systemic sclerosis (SSc), dermatomyositis, and polymyositis (DM/PM), undifferentiated connective tissue disorders (UDCTDs), and overlap syndromes such as mixed connective tissue disorders (MCTDs) [2]. Immune disorders can affect every organ in the human body, including the central and peripheral neurological systems [3]. Peripheral neuropathy, which can manifest as either confluent and symmetrical or multifocal and asymmetrical, is a well-known complication of CTDs. Vasculitis, characterized by immune-mediated changes in the blood vessel walls, can manifest with symptoms affecting both the central and peripheral nervous systems [4]. These vascular injuries are often linked to the presence of various autoantibodies.

While SS is a common association, other relevant autoantibodies include antinuclear antibodies (ANAs), extractable nuclear antigen antibodies (Sjögren's syndrome antigen A (SSA)/Sjögren's syndrome antigen B (SSB)), rheumatoid factor (RF), anticardiolipin antibodies (ACAs), cryoglobulins, and anti-double-stranded DNA antibodies (dsDNA) [5,6]. The involvement of these antibodies contributes to the inflammatory process, leading to vascular damage and subsequent neurological symptoms [7]. Peripheral neuropathy is a frequent neurologic issue in immune-mediated rheumatic diseases, notably affecting up to 60% of SS patients, 15% of those with SLE, and 70% of those with systemic vasculitis [8]. Early detection of peripheral neuropathy in patients with AICTD is challenging because nerve conduction studies are not routinely performed [9]. Identifying and managing this condition early could alleviate the burden of peripheral neuropathy among AICTD patients. There is a lack of indicators to guide physicians on when to conduct nerve conduction studies to detect early peripheral neuropathy in these patients. In this context, a study was conducted to determine the prevalence of subclinical peripheral neuropathy among AICTD patients and to assess its association with potential predictor variables.

## Materials and methods

Study design and study population

This research was an observational cross-sectional study. The study included a total of 100 patients who had been undergoing treatment for AICTDs for three or more years.

Study duration

The study was conducted over a period of 15 months (June 1, 2021, to August 31, 2022).

Ethical clearance

Ethical approval was obtained from the Institutional Ethics Committee before the study commenced. The Ethical Committee approval number was SMC/IEC/2020/12/045.

Inclusion criteria

Inclusion criteria included all patients with AICTD who were not on immunosuppressants (other than steroids) for a period of three or more years, with informed consent for the research.

Exclusion criteria

Patients with established peripheral neuropathy, patients taking any immunosuppressive drug (other than steroids), and patients with diseases like diabetes mellitus, malignancy, and psychiatric disorders were excluded from the study. AICTD patients with thyroid disorders, those with alcoholic or nutritional deficiencies, drugs causing peripheral neuropathy, known malignancy, and infections like HIV and leprosy were excluded. However, vasculitis, being a part of the underlying disease process of the study population, was not excluded. AICTD patients on disease-modifying antirheumatic drugs (DMARDs) were excluded from the study. Similarly, pregnant and lactating women were excluded from our study.

Sample size

In a 2018 study by Perzyńska-Mazan et al. involving patients with primary Sjögren's syndrome (PSS), an AICTD, it was found that neurological symptoms were present in approximately 8.5% to 70% of the patients [10]. According to the 2018 study by Perzyńska-Mazan et al., using a prevalence of 50% and an absolute error of 10%, the minimum sample size required is 100. This is calculated using the formula 3.84*p*q*/d^2, where p is prevalence, q is the complement of p, and d is the precision.

Sampling technique

The non-probability convenience sampling method was applied to select the samples for this study. The first 100 patients diagnosed in our tertiary care hospital as having AICTD during the study period (those who were willing to participate in the study) were included as study samples.

Data collection

All data were gathered by a single investigator through direct interviews and clinical examinations. Subclinical peripheral neuropathy in these patients was identified using nerve conduction studies, which were conducted on multiple nerves, including the bilateral ulnar, radial, sural, peroneal, and tibial nerves, at a tertiary-level medical college hospital. Ethical approval was obtained, and informed consent was provided by all patients before the procedures. In addition to nerve conduction studies, the patients underwent various laboratory tests, including hemoglobin, total count, differential count, and erythrocyte sedimentation rate (ESR). Important biomarkers, such as C-reactive protein (CRP), RF status, and anti-cyclic citrullinated peptide (Anti-CCP) antibody status, were also evaluated to provide a comprehensive assessment of their condition.

Statistical analysis

Descriptive statistics were utilized to calculate the prevalence of different types of neuropathies within the study population. Data from nerve conduction studies were analyzed using Chi-square or Fisher’s exact tests to compare neuropathic changes. Comparative analysis was performed through independent samples t-tests. Logistic regression was used to identify factors predisposing to neuropathy. All analyses were performed at a 0.05 significance level using IBM SPSS Statistics for Windows, Version 20 (Released 2011; IBM Corp., Armonk, NY, USA).

## Results

A total of 100 subjects were enrolled in the study. Table 1 outlines the baseline characteristics of the study population. The mean age of the participants is 43.8 years. The majority of the study population was female, comprising 79 (79%).

**Table 1:** Baseline characteristics of the study participants (n = 100)

Baseline characteristics		Frequency (n)	Percentage (%)
Age in years		Mean: 43.8, Standard deviation: 16.9	
Gender	Male	21	21%
	Female	79	79%

Table 2 details the subclinical peripheral neuropathy status of the study population. The prevalence of subclinical peripheral neuropathy among the participants is 18 (18%).

**Table 2:** Subclinical peripheral neuropathy in the study subjects (n = 100)

Status	Frequency (n)	Percentage (%)
Peripheral neuropathy present	18	18%
Peripheral neuropathy absent	82	82%

Table 3 details the association between demographic parameters and the presence of subclinical peripheral neuropathy. The study results indicate that participants with subclinical peripheral neuropathy had a mean age of 49.66 ± 4.77 years, while those without it had a mean age of 46.02 ± 12.7 years, with a statistically significant difference determined by a t-test (p-value = 0.047). The gender distribution was not significantly different between the groups: 27.8% of those with neuropathy were male, and 72.2% were female, compared to 20.7% male and 79.3% female in the group without neuropathy, as shown by a Chi-square test (p-value = 0.513).

**Table 3:** Association between demographic parameters and presence of subclinical peripheral neuropathy among the study participants (n = 100) *p-value less than 0.05 is considered significant

Variables		Subclinical peripheral neuropathy		Total	p-value*
		Yes (%)	No (%)		
Age in years (mean ± SD)		49.66 ± 4.77	46.02 ± 12.7	100	0.047*
Gender	Male	05 (27.8)	17 (20.7)	22	0.513
	Female	13 (72.2)	65 (79.3)	78	

Table 4 presents the association between laboratory parameters and the presence of subclinical peripheral neuropathy among the study participants. The mean hemoglobin levels were similar between participants with and without neuropathy (11.48 ± 1.75 vs. 12.08 ± 2.7), with no statistically significant difference, as determined by a t-test (p-value = 0.880). The ESR also showed no significant difference (33.22 ± 14.04 vs. 31.26 ± 12.52, p-value = 0.559). Total leukocyte count was higher in those without neuropathy (10221.9 ± 2403.9 vs. 9133.3 ± 2377.1), approaching significance (p-value = 0.084). Other parameters, including polymorphs, lymphocytes, monocytes, and platelets, also showed no significant differences between the groups (p-values = 0.164, 0.705, 0.381, and 0.923, respectively), as assessed by t-tests.

**Table 4:** Association between laboratory parameters and presence of subclinical peripheral neuropathy among the study participants (n = 100) *p-value less than 0.05 is considered significant

Variables	Subclinical peripheral neuropathy		p-value*
	Yes	No	
Hemoglobin (mean ± SD)	11.48 ± 1.75	12.08 ± 2.7	0.880
Erythrocyte sedimentation rate (mean ± SD)	33.22 ± 14.04	31.26 ± 12.52	0.559
Total leucocyte count (mean ± SD)	9133.3 ± 2377.1	10221.9 ± 2403.9	0.084
Polymorphs (mean ± SD)	61.77 ± 4.18	63.17 ± 3.73	0.164
Lymphocyte (mean ± SD)	29.11 ± 5.23	28.70 ± 3.80	0.705
Monocyte (mean ± SD)	3.13 ± 0.514	2.99 ± 0.622	0.381
Platelets (mean ± SD)	2.65 ± 0.47	2.65 ± 0.483	0.923

Table 5 displays the association between autoimmune markers and the presence of subclinical peripheral neuropathy among the study participants. For CRP, there was no significant difference in positivity between those with and without neuropathy (61.1% vs. 58.5%, p-value = 0.841). However, for RF, a significantly higher proportion of participants with neuropathy tested positive compared to those without neuropathy (66.7% vs. 58.5%, p-value = 0.011). Similarly, for Anti-CCP antibody status, a significantly higher percentage of participants with neuropathy tested positive compared to those without neuropathy (78.5% vs. 47%, p-value = 0.032). These associations were determined using Chi-square tests.

**Table 5:** Association between autoimmune markers between and presence of subclinical peripheral neuropathy among the study participants (n = 100) *p-value less than 0.05 is considered significant

Marker	Status	Subclinical peripheral neuropathy		p-value*
		Yes, n (%)	No, n (%)	
C-reactive protein	Positive	11 (61.1)	48 (58.5)	0.841
	Negative	07 (38.9)	34 (41.5)	
Rheumatoid factor status	Positive	08 (66.7)	71 (58.5)	0.011*
	Negative	04 (33.3)	11 (13.4)	
Anti-CCP antibody status	Positive	11 (78.5)	32 (47)	0.032*
	Negative	03 (21.5)	36 (53)	

Table 6 presents the association between steroid use and the presence of subclinical peripheral neuropathy among the study participants. Among those with subclinical peripheral neuropathy, 16% were on steroid medication compared to 84% who were not, while 20% of those without neuropathy were on steroids, compared to 80% who were not. This difference was not statistically significant, as determined by a Chi-square test (p-value = 0.769).

**Table 6:** Association between steroid use and presence of subclinical peripheral neuropathy among the study participants (n = 100) *p-value less than 0.05 is considered significant

Steroid medication status	Subclinical peripheral neuropathy		p-value*
	Yes, n (%)	No, n (%)	
Yes	8 (16)	42 (84)	0.769
No	10 (20)	40 (80)	

## Discussion

AICTDs can impact both the central and peripheral nervous systems [11]. The two most common types of peripheral nervous system involvement are symmetric sensorimotor peripheral neuropathy and symmetric pure-sensory peripheral neuropathy (PSPN) [12,13]. We recruited 100 subjects for our study, with the majority being female, comprising approximately 79% of the participants. This indicates a higher prevalence of AICTDs among females compared to males. Fairweather et al. found similar results in their research. According to Fairweather et al., 78% of individuals with AICTDs, which affect all organs and systems, are women [12]. In our study, 79% of the participants were female. Previous research indicates that women produce more immunoglobulins, exhibit greater immunoreactivity, and generate more antibodies in response to antigen stimulation compared to men [14]. Estrogen and prolactin are pro-inflammatory hormones, and higher exposure to these hormones explains the high female-to-male ratio.

In our study, 18 out of 100 patients experienced peripheral neuropathy, accounting for 18% of those with AICTDs. Of these 18 individuals, 13 were female, representing approximately 72%. Dani et al. reported that 10% of patients had clinical signs of peripheral neuropathy, while 50% had subclinical neuropathy [15]. Bharadwaj and Haroon found that 8.57% of patients with rheumatoid arthritis developed neuropathy [16]. Participants with peripheral neuropathy had a mean age of 49.66 ± 4.77 years, compared to 46.02 ± 12.7 years for those without neuropathy, a statistically significant difference (p = 0.047). Of patients with peripheral neuropathy, none were under 40 years old; 16 were between 40 and 60 years old, and two were over 60 years old.

Our study found no association between inflammatory markers like ESR, CRP, and subclinical peripheral neuropathy. This contrasts with a study by Kaeley et al., which observed a correlation between peripheral neuropathy and inflammatory markers of disease activity, specifically CRP and ESR [17]. This difference needs further research.

Our study found that about 66.7% of participants with subclinical neuropathy tested positive for RF, compared to 58.5% of subjects without neuropathy. Statistical analysis revealed a significant difference (p = 0.011), indicating a higher prevalence of peripheral neuropathy among patients with a positive RF. Similar findings have been reported in studies by Albani et al. and Biswas et al. [18,19]. Additionally, among patients with subclinical neuropathy, approximately 88.3% tested positive for Anti-CCP antibodies, whereas only 54.9% of patients without peripheral neuropathy tested positive. This difference was also statistically significant (p = 0.026). Sim et al. have identified a connection between Anti-CCP antibodies and a heightened risk of peripheral neuropathy [20]. Although there are few studies discussing the association between peripheral neuropathy and AICTD, this finding provides a novel hypothesis for further research [18,19].

Our study found no statistical significance with steroid use for peripheral neuropathy. Contrary to findings by Lanzillo et al., who reported a protective effect of prior corticosteroid use on the development of peripheral neuropathy, our study reveals opposing results [21]. This difference might require further research.

Limitations

This study was conducted in a single tertiary care hospital. A multi-centric study involving more subjects could yield more reliable results. Additionally, the duration of AICTDs in the study population, set at more than three years, appears arbitrary. Further research with larger sample sizes, a multi-centric approach, and more precise criteria for disease duration is warranted to provide more reliable insights into these findings.

## Conclusions

The study identified a notable prevalence of subclinical peripheral neuropathy (18%) among individuals with AICTDs, detectable only through nerve conduction studies. Interestingly, gender did not significantly influence the likelihood of developing neuropathy, as the distribution was similar across those with and without the condition. Additionally, key laboratory parameters, including hemoglobin levels, ESR, and total leukocyte count, did not show significant differences between the groups, suggesting that these factors might not be reliable indicators for neuropathy in this patient population. Steroid use also demonstrated no significant correlation with the presence of subclinical peripheral neuropathy. However, the study found that autoimmune markers, such as RF and Anti-CCP antibodies, were significantly associated with the condition, highlighting their potential as indicators for early detection. These findings underscore the importance of considering nerve conduction studies in patients with these markers, as early identification of subclinical neuropathy could lead to more timely interventions. The study also opens the door for future research to explore whether more aggressive treatment strategies could help reduce the occurrence of peripheral neuropathy by targeting and managing these subclinical cases early on.
